# Supplementary material for: An integrated multi-omics analysis identifies prognostic molecular subtypes of non-muscle-invasive bladder cancer
Source: Nat Commun. 2021 Apr 16;12:2301. doi: 10.1038/s41467-021-22465-w (PMC8052448; doi:10.1038/s41467-021-22465-w)
Supplement: Supplementary file 1 — Supplementary Information [file 41467_2021_22465_MOESM1_ESM.pdf]

Supplementary Information for

An integrated multi-omics analysis identifies prognostic molecular subtypes of non-muscle-invasive bladder cancer

Lindskrog and Prip *et al.*

Supplementary Information

Supplementary Figures **2**

Supplementary Figure 1 2

Supplementary Figure 2 3

Supplementary Figure 3 4

Supplementary Figure 4 5

Supplementary Figure 5 6

Supplementary Figure 6 7

Supplementary Figure 7 8

Supplementary Figure 8 9

Supplementary Figure 9 10

Supplementary Tables **11**

Supplementary Table 1 11

Supplementary Table 2 12

Supplementary Table 3 15

Supplementary Table 4 16

Supplementary Table 5 17

Supplementary Table 6 18

Supplementary Table 7 19

Supplementary Table 8 20

Supplementary Table 9 21

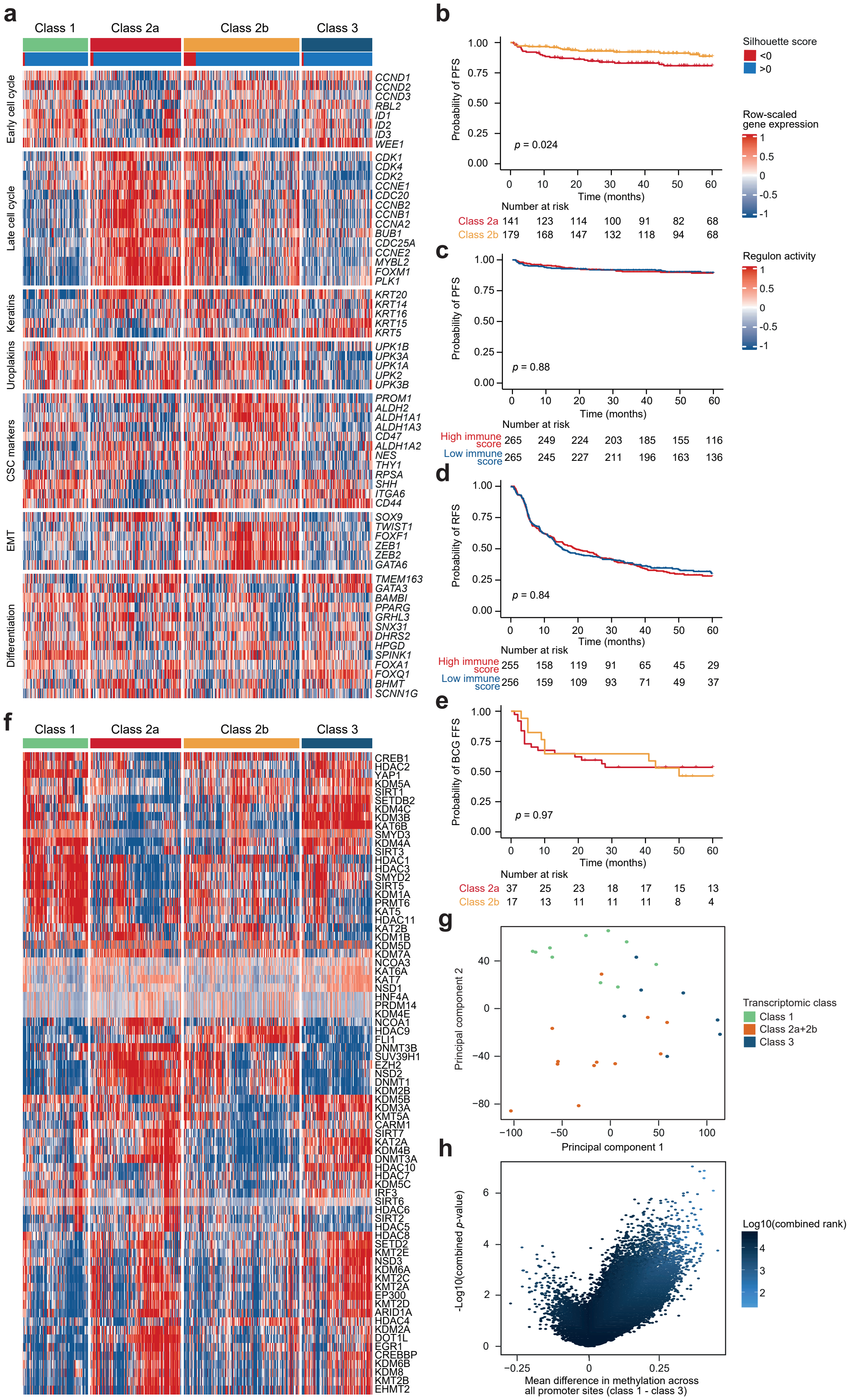

Supplementary Figure 1. Gene expression, regulons and methylation of transcriptomic classes. a Expression matrix of genes in selected biological processes. Samples are ordered after increasing silhouette score within each transcriptomic class (lowest to highest class correlation). CSC = cancer stem cell; EMT = epithelial-mesenchymal transition. **b** Kaplan-Meier plot of progression-free survival (PFS) for class 2a and 2b patients. **c** Kaplan-Meier plot of PFS for patients stratified by RNA-based immune score (above or below median). **d** Kaplan-Meier plot of recurrence-free survival (RFS) for patients stratified by RNA-based immune score (above or below median). **e** Kaplan-Meier plot of BCG failure-free survival (FFS) for patients stratified by transcriptomic class. **f** Regulon activity profiles for potential regulators associated with chromatin remodeling. **g** Principal component analysis of DNA methylation data from 29 Ta tumors representing the different transcriptomic classes. **h** Volcano plot of the mean promoter methylation difference between class 1 (n=10) and class 3 (n=7) on the site level. The combined *p*-value was computed by combining *p*-values of all sites in the region using the Fisher's method. The comparison is colored according to the combined ranks (mean difference, mean quotient and FDR adjusted *p*-value). Two-sided log-rank tests were used to compare survival curves. Source data are provided as a Supplementary Source Data file.

a

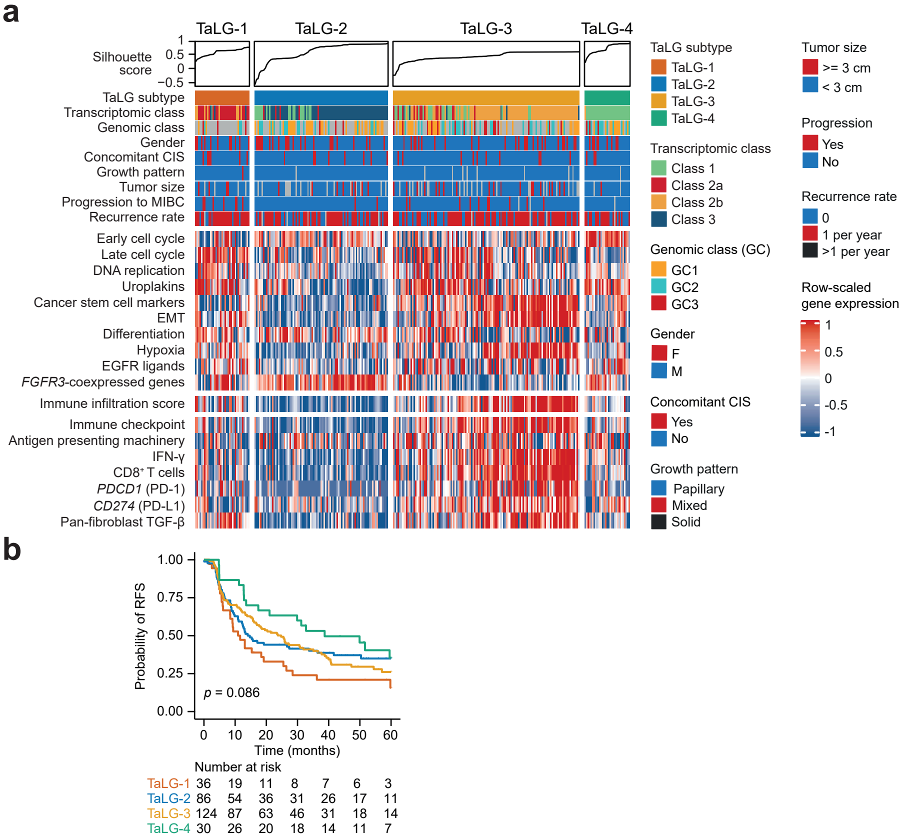

Supplementary Figure 2. Ta low grade subtypes. a Clinicopathological information and selected gene expression signatures for all Ta low grade (TaLG) tumors (n=286) stratified by TaLG subtype. Samples are ordered after increasing silhouette score within each subtype (lowest to highest subtype correlation). **b** Kaplan-Meier plot of recurrence-free survival (RFS) for 276 patients stratified by TaLG subtype. A two-sided log-rank test was used to compare survival curves. CIS = carcinoma *in situ*; MIBC = muscle-invasive bladder cancer; EMT = epithelial-mesenchymal transition. Source data are provided as a Supplementary Source Data file.

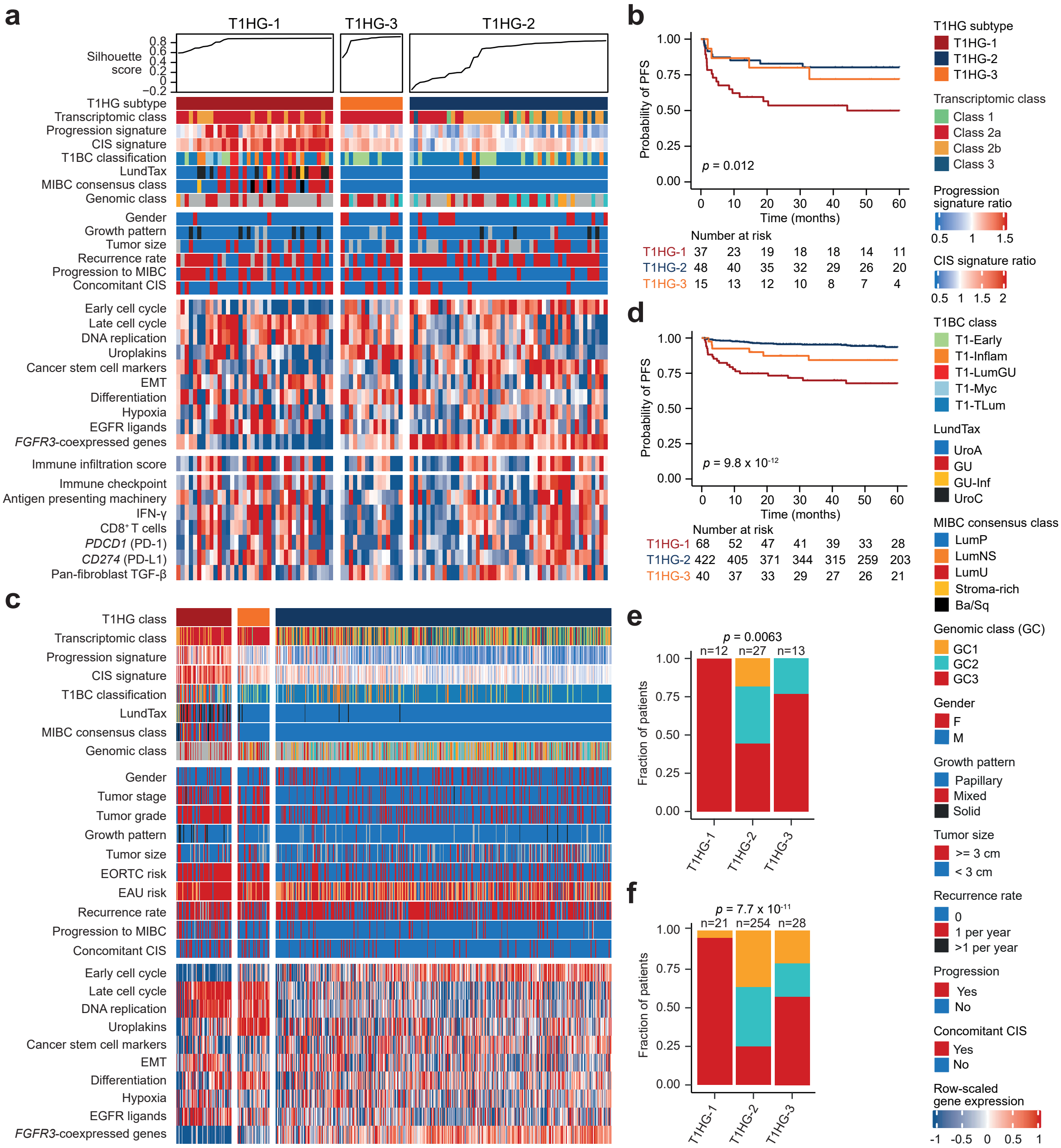

Supplementary Figure 3. T1 high grade subtypes. **a** Clinicopathological information and selected gene expression signatures for all T1 high grade (T1HG) tumors (n=101) stratified by T1HG subtype. Samples are ordered after increasing silhouette score within each subtype (lowest to highest subtype correlation). **b** Kaplan-Meier plot of progression-free survival (PFS) for 100 patients stratified by T1HG subtype. **c** Clinicopathological information and selected gene expression signatures for all tumors (n=535) stratified by T1HG subtype. **d** Kaplan-Meier plot of PFS for 530 patients stratified by T1HG subtype. **e** Genomic classes (GCs) compared to T1HG subtypes (n=52, all T1HG tumors). **f** Genomic classes (GCs) compared to T1HG subtypes (n=303, all tumors). *P*-values were calculated using two-sided Fisher's exact test for categorical variables and two-sided log-rank test for comparing survival curves. CIS = carcinoma *in situ*; MIBC = muscle-invasive bladder cancer; EMT = epithelial-mesenchymal transition. Source data are provided as a Supplementary Source Data file.

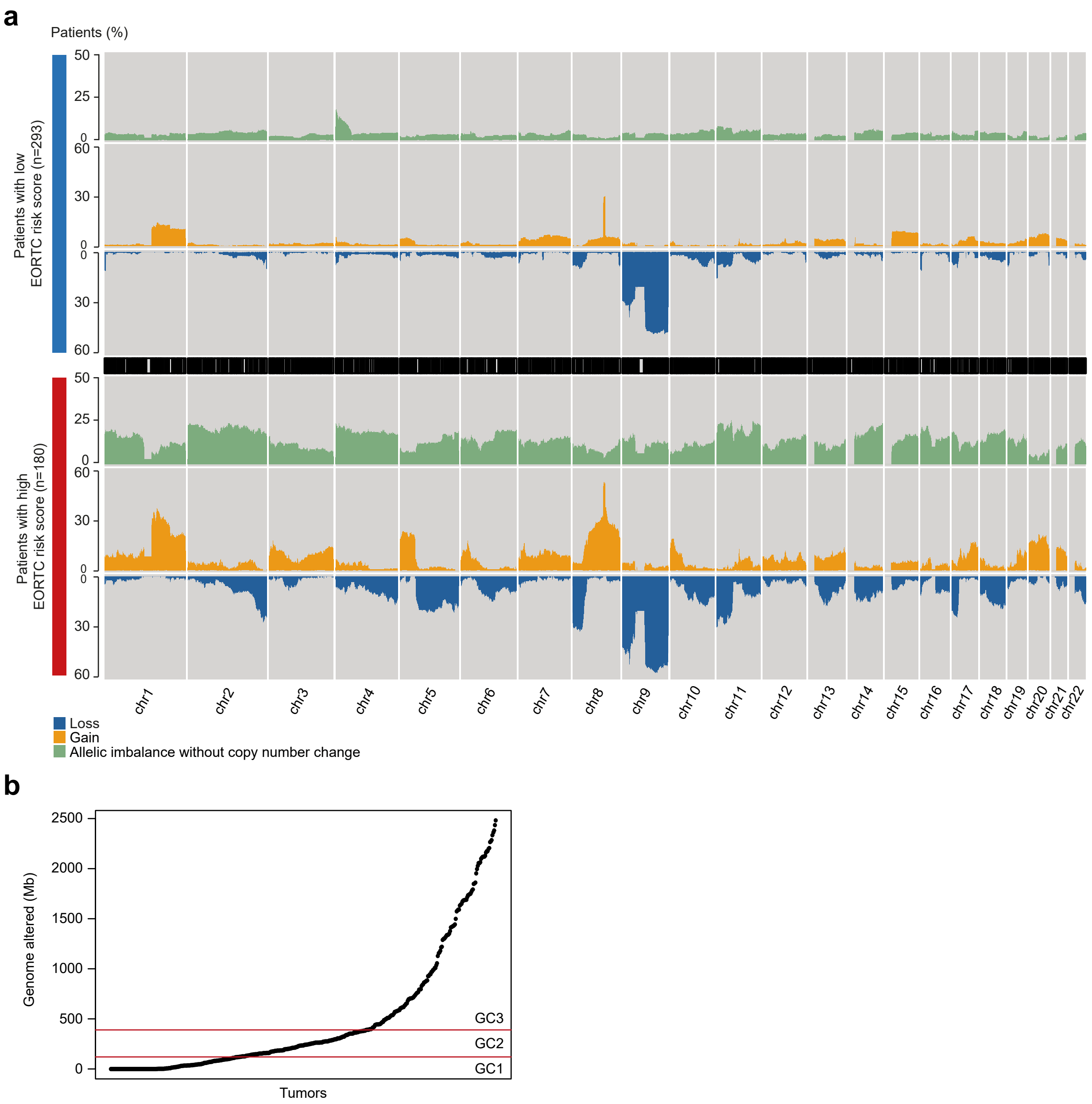

Supplementary Figure 4. Copy number alterations in NMIBC. a Genomic landscape of gains, losses and allelic imbalance without copy number change according to high (>6) and low (≤6) EORTC risk scores. **b** Amount of genomic alterations (Mb) according to genomic classes (GCs). Tumors were stratified into three GCs of equal size with increasing copy number alteration burden to illustrate low, intermediate and high chromosomal instability. EORTC = European Organisation for Research and Treatment of Cancer. Source data are provided as a Supplementary Source Data file.

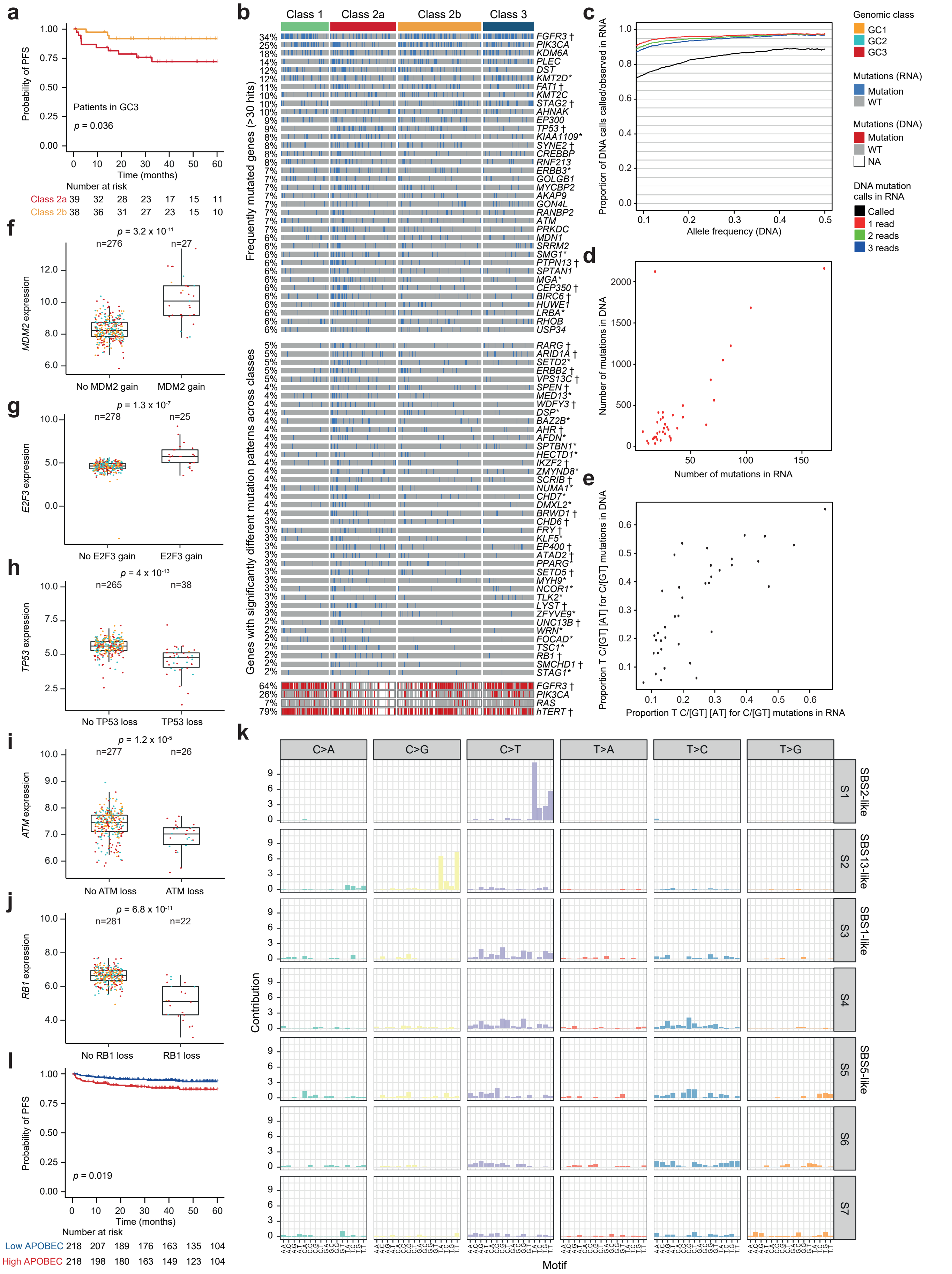

Supplementary Figure 5. Genomic alterations associated with transcriptomic classes. a Kaplan-Meier plot of progression-free survival (PFS) for patients in genomic class (GC) 3 stratified by transcriptomic class. **b** RNA-derived mutations (top panels) and DNA-derived hot-spot mutations (bottom panel). Samples are ordered after the combined contribution of the APOBEC-related mutational signatures. Asterisks indicate *p*-values <0.05. Daggers indicate BH-adjusted *p*-values <0.05. **c** Proportion of DNA mutations calls observed or called in RNA for 38 patients. **d** Correlation between the number of mutations in DNA (VAF>0.1) and RNA for 38 patients. Only the 791 genes used to calculate the RNA-derived mutational load were considered for the RNA-derived mutations. **e** Proportion of all C to T/G mutations in a TCW context in DNA vs. RNA (proxy for APOBEC-related mutations). **f-j** Gene expression of key molecules in the p53 pathway stratified by gene copy number status. **k** Contribution of each mutation type defined by the substitution class (top) and the flanking 5' and 3' nucleotides (bottom). Only tumors with >100 single-nucleotide variations (n=441) were used to infer the seven mutational signatures. **l** Kaplan-Meier plot of PFS for patients stratified by contribution of the APOBEC-related signatures (above or below median). Statistical significance was assessed using a two-sided Fisher's exact test for categorical variables, a two-sided Wilcoxon rank sum test for continuous variables and a two-sided log-rank test for comparing survival curves. For all boxplots, the center line represents the median, box hinges represent first and third quartiles and whiskers represent ±1.5 x interquartile range. Source data are provided as a Supplementary Source Data file.

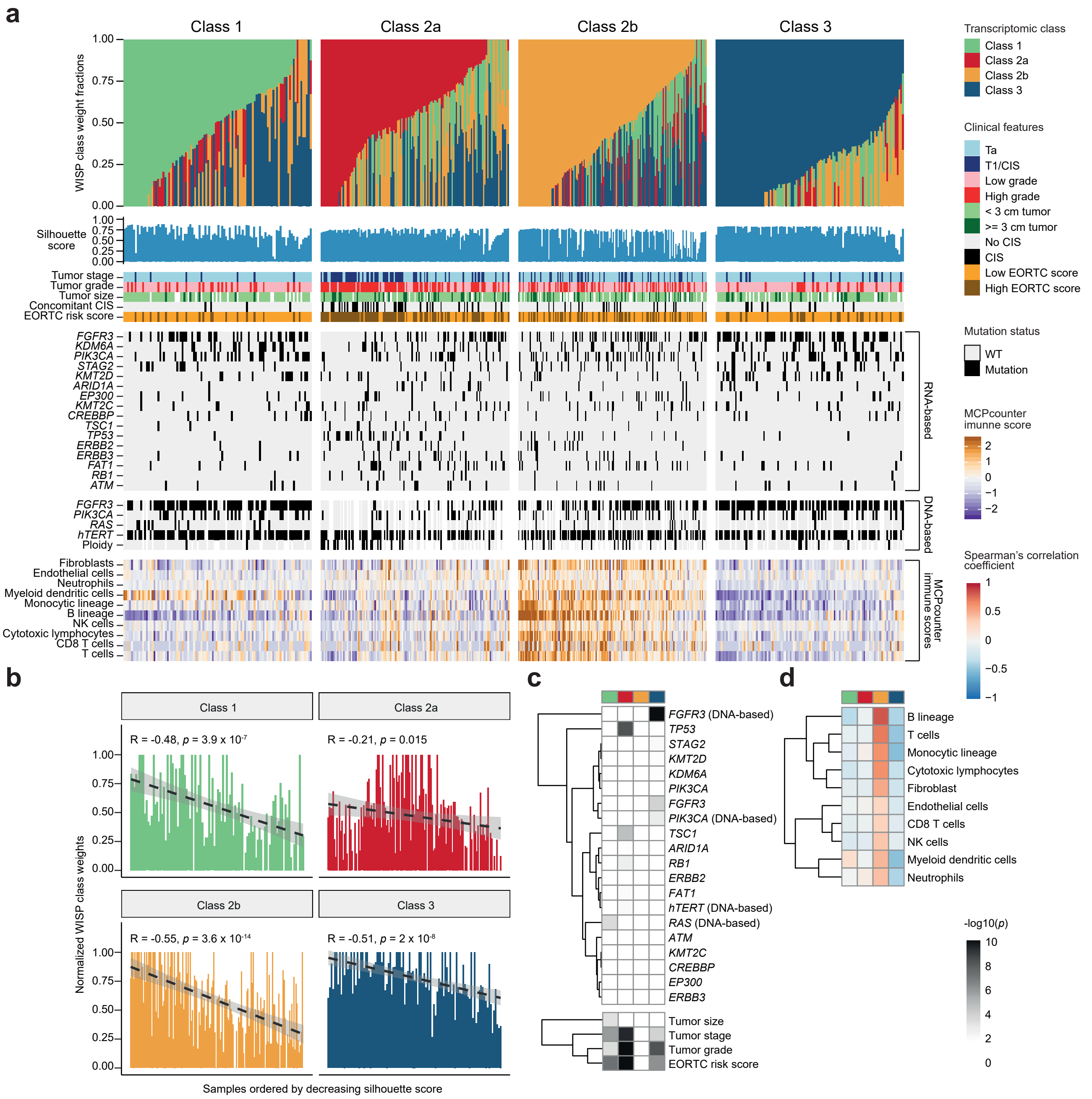

Supplementary Figure 6. Assessment of intra-tumoral heterogeneity using Weighted In-Silico Pathology (WISP) class weights. **a** Semi-supervised visualization of WISP class weights. Samples are ordered by decreasing WISP class weights within the respective classes assigned by consensus clustering. Panels: Proportion of WISP class weights; silhouette scores; clinical and histopathological features; RNA-based mutations; DNA-based mutations and ploidy; MCPcounter immune and stromal population scores. **b** Correlation between silhouette scores from consensus clustering and WISP class weights for each class. Correlation coefficients with *p*-values were calculated from Pearson's correlation tests. Shaded areas indicate 95% confidence intervals for the fitted linear regression curves. **c** Association between WISP class weights and mutations (top) and clinical features (bottom). *P*-values were calculated using two-sided Wilcoxon rank sum test. **d** Spearman's correlation of WISP class weights and MCPcounter immune and stromal scores. CIS = carcinoma *in situ*; EORTC = European Organisation for Research and Treatment of Cancer. Source data are provided as a Supplementary Source Data file.

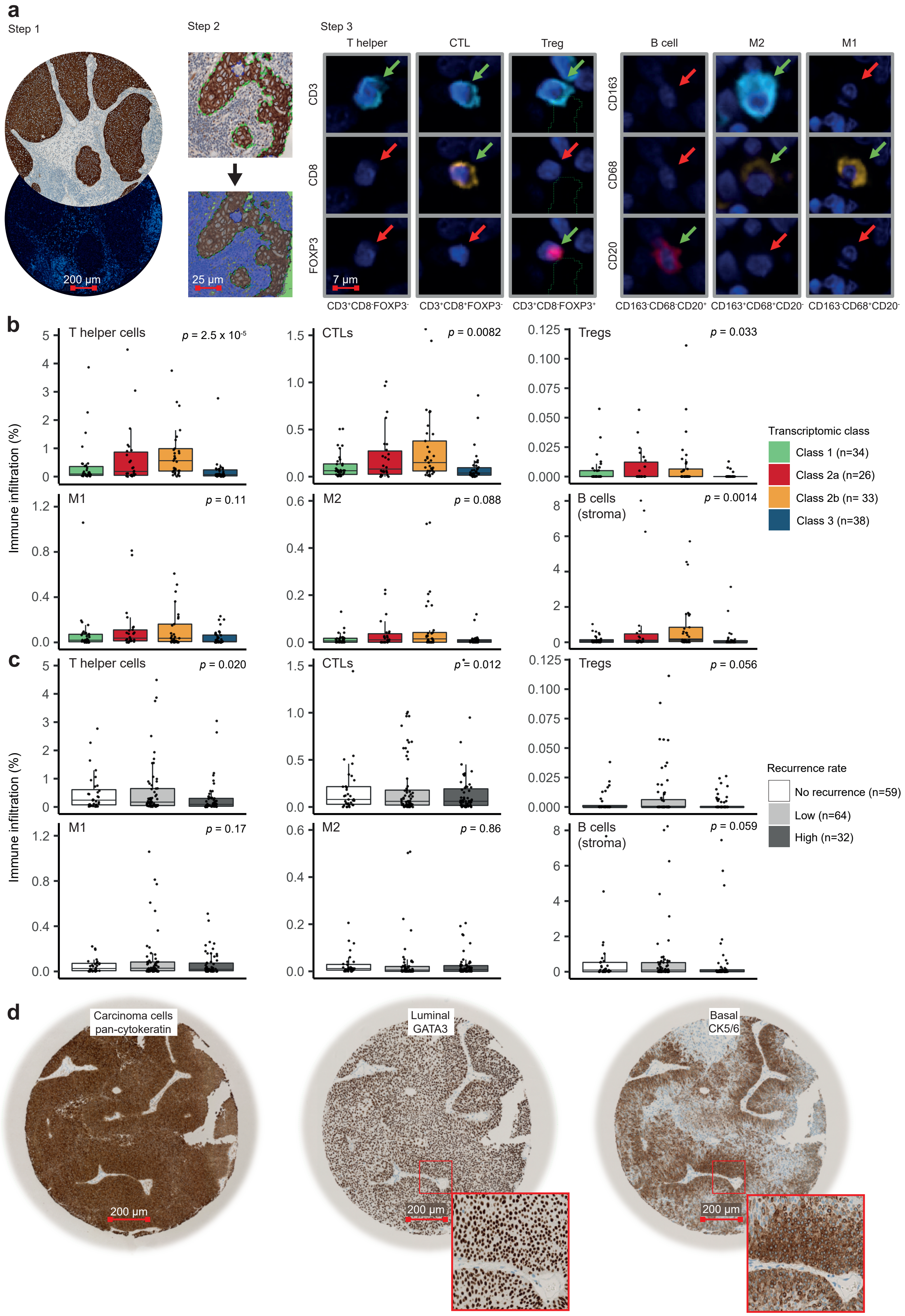

Supplementary Figure 7. Spatial proteomics analysis of the tumor immune contexture. a The digital pathology workflow. Step 1: Alignment of the pan-cytokeratin staining with the multiplex immunofluorescence staining. Step 2: Automated detection of the tumor parenchyma and stroma. Step 3: Automated detection of immune cells based on co-expression. All protein measurements were performed once for each distinct sample. **b** Immune cell infiltration according to transcriptomic class. Immune infiltration is defined as the percentage of cells in the parenchyma classified as the different immune cells. B cells were quantified in the stroma due to a low infiltration. *P*-values were calculated using a two-sided Wilcoxon rank sum test. **c** Immune cell infiltration according to recurrence rate. *P*-values were calculated by the one-sided Jonckheere-Terpstra test for trend. **d** Illustration of a tumor with both luminal (GATA3) and basal (CK5/6) characteristics. All protein measurements were performed once for each distinct sample. For all boxplots, the center line represents the median, box hinges represent first and third quartiles and whiskers represent ±1.5 x interquartile range. CTLs = cytotoxic T lymphocytes; Tregs = regulatory T cells. Source data are provided as a Supplementary Source Data file.

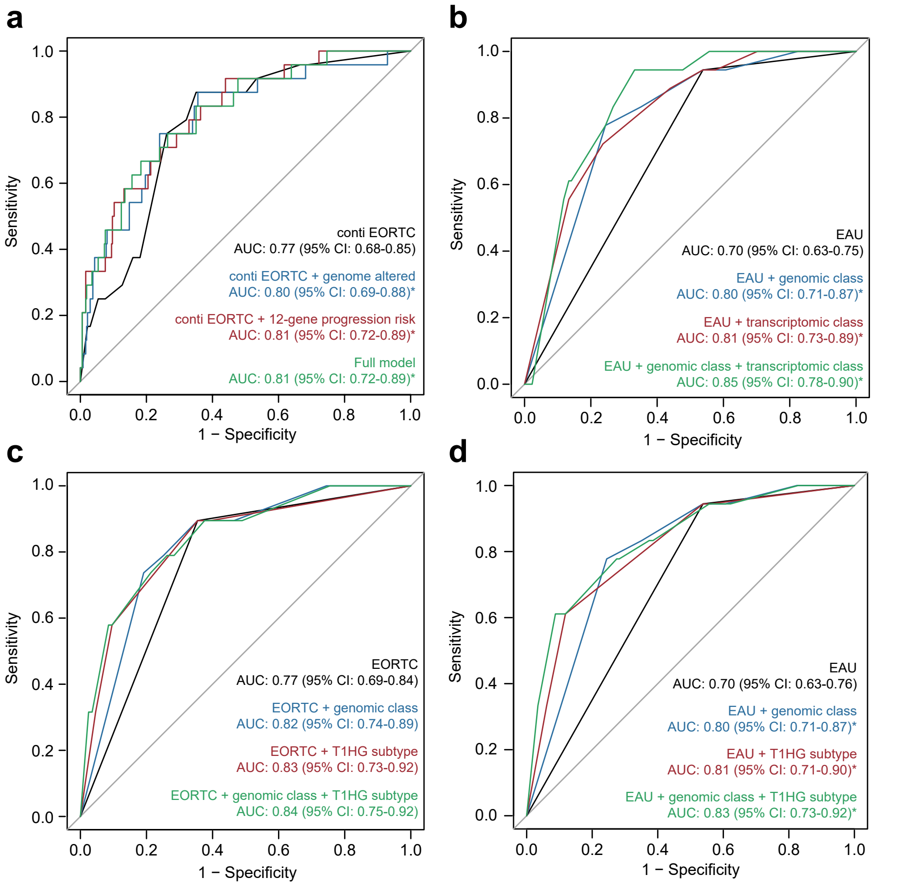

Supplementary Figure 8. Prediction models. a Receiver operating characteristic (ROC) curves for predicting progression within 5 years using logistic regression models with continuous (conti) variables (n=395, events=24). **b** ROC curves for predicting progression within 5 years using logistic regression models including EAU risk score, genomic class and transcriptomic class (n=280, events=18). **c** ROC curves for predicting progression within 5 years using logistic regression models including EORTC risk score, genomic class and T1HG subtype (n=301, events=19). **d** ROC curves for predicting progression within 5 years using logistic regression models including EAU risk score, genomic class and T1HG subtype (n=280, events=18). Asterisks indicate significant improvement compared to the EORTC/EAU model (Likelihood ratio test, BH-adjusted *p*-value below 0.05). EORTC = European Organisation for Research and Treatment of Cancer; EAU = European Association of Urology; AUC = area under the curve; CI = confidence interval. Source data are provided as a Supplementary Source Data file.

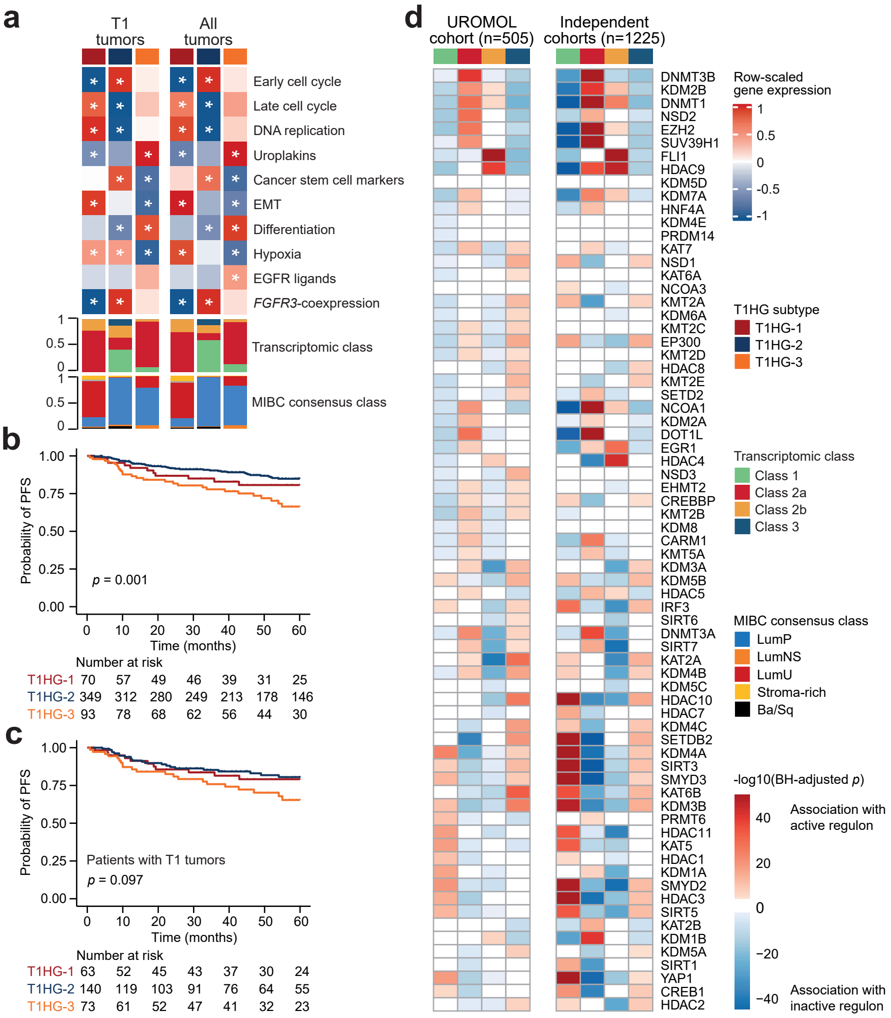

Supplementary Figure 9. Validation of transcriptomic classes in independent cohorts. **a** Mean expression of selected gene expression signatures and proportion of transcriptomic UROMOL2021 and MIBC consensus classifications for T1HG classifications of T1 tumors (n=663) and all tumors (n=1226) in the independent cohorts. Asterisks indicate significant association between gene expression signature and subtype (one subtype vs. all other subtypes, two-sided Wilcoxon rank sum test, BH-adjusted *p*-value below 0.05). EMT = epithelial-mesenchymal transition; MIBC = muscle-invasive bladder cancer. **b** Kaplan-Meier plot of progression-free survival (PFS) for 512 patients from independent cohorts stratified by T1HG subtype. **c** Kaplan-Meier plot of PFS for 276 patients with T1 tumors from independent cohorts stratified by T1HG subtype. **d** Association of epigenetic-driven regulon activities (active vs. repressed status) with transcriptomic UROMOL2021 classes in the UROMOL cohort (including samples with positive silhouette scores) and in the independent cohorts (pooled). The heatmap illustrates BH-adjusted *p*-values from two-sided Fisher's exact tests. Two-sided log-rank tests were used to compare survival curves.Source data are provided as a Supplementary Source Data file.

Supplementary Table 1. Clinical characteristics and multi-omics platforms

	Copy number analysis (n=473)	RNA-Sequencing (n=535)	12-gene qPCR (n=735)	Multiplex IF and IHC (n=167)	Total (n=834)
Median follow up (years)	3.51	4.55	3.62	4.89	3.89
Median follow up for non-progressors (years)	3.62	4.76	3.87	5.01	4.12
Median age (years)	70	69	70	69	70
Gender					
Male	354	414	568	138	644
Female	119	121	167	29	190
Smoking status					
Current	162	169	221	76	238
Former	150	167	242	68	257
Never	70	62	96	19	101
Unknown	91	137	176	4	238
Stage					
Ta	335	397	523	117	592
T1	131	135	201	50	231
CIS	7	3	11	0	11
Grade					
High	199	215	305	77	353
Low	274	320	430	90	481
Concomitant CIS					
No	408	458	630	145	723
Yes	65	77	105	22	111
Growth pattern					
Papillary	422	480	627	161	710
Mixed	7	10	16	1	21
Solid	7	13	16	1	23
Unknown	37	32	76	4	80
Size					
< 3 cm	294	346	441	107	510
≥ 3 cm	67	90	113	46	131
Unknown	112	99	181	14	193
Incident tumor					
Yes	247	287	393	91	457
No	226	248	342	76	377
Progression to MIBC					
Yes	35	65	54	14	78
No	391	465	606	146	677
No follow-up	47	5	75	7	79
Recurrence					
Yes	239	348	339	104	410
No	160	163	198	56	219
No follow-up	74	24	198	7	205
Median recurrence rate per year	0.24	0.33	0.33	0.33	0.30
EORTC risk score					
High (>6)	179	211	284	76	326
Low (≤6)	294	324	451	91	508

IF = immunofluorescence; IHC = immunohistochemistry; CIS = carcinoma in situ; EORTC = European Organisation for Research and Treatment of Cancer. Source data are provided as a Supplementary Source Data file.

Supplementary Table 2. Cox regression analysis

Progression-free survival		
	HR (95% CI)	*p*-value
Univariate analysis		
Clinical features (n=755, 78 events)		
Age (n=747, 78 events)	1.24 (1.11-1.39)	0.0001
Gender (male vs female)	1.31 (0.75-2.31)	0.35
Stage (T1/CIS vs Ta)	4.09 (2.59-6.42)	1.1×10^{-9}
Grade (high vs low)	3.37 (2.10-5.41)	4.4×10^{-6}
Concomitant CIS (yes vs no)	1.04 (0.55-1.96)	0.91
EORTC risk score (>6 vs ≤6)	4.12 (2.55-6.67)	8.4×10^{-9}
EAU risk score (High vs. intermediate/low; n=708, 74 events)	3.34 (1.92-5.83)	2.2×10^{-5}
Smoking history (Current/former vs never; n=556, 56 events)	0.90 (0.45-1.79)	0.77
RNA-Seq features(n=530, 65 events)		
Transcriptomic class 2a vs 1	6.92 (2.11-22.72)	0.001
Transcriptomic class 2b vs 1	4.29 (1.29-14.31)	0.018
Transcriptomic class 3 vs 1	1.88 (0.48-7.27)	0.36
Progression signature (continuous)	7.30 (3.52-15.15)	9.6×10^{-8}
CIS signature (continuous)	2.51 (1.75-3.60)	6.5×10^{-7}
T1HG subtype 1 vs 2	4.71 (2.76-8.04)	1.4×10^{-8}
T1HG subtype 3 vs 2	2.64 (1.22-5.73)	0.014
Immune score (continuous)	1.00 (0.98-1.02)	0.98
Mutational signatures (n=436, 52 events)		
Signature 1 (above vs below median)	0.81 (0.47-1.40)	0.46
Signature 2 (above vs below median)	1.48 (0.85-2.58)	0.16
Signature 5 (above vs below median)	0.05 (0.63-1.86)	0.79
Signature 13 (above vs below median)	2.12 (1.20-3.76)	0.01
qPCR features (n=660, 54 events)		
Progression signature (high vs low)	3.97 (2.13-7.42)	1.5×10^{-5}
Progression signature (continuous)	2.10 (1.68-2.61)	3.2×10^{-11}
Copy number features (n=426, 35 events)		
Genomic classes (GC3 vs GC1/2)	4.26 (2.17-8.59)	5.0×10^{-5}
Genome altered in Mb (continuous)	3.05 (2.01-4.62)	1.4×10^{-7}
Multivariable model 1 (n=426, 35 events)		
EORTC risk score (>6 vs ≤6)	2.73 (1.24-6.00)	0.0127
Genomic classes (GC3 vs GC1/2)	2.76 (1.28-5.97)	0.0098
Multivariable model 2 (n=393, 31 events)		
EAU risk score (High vs intermediate/low)	3.33 (1.12-9.92)	0.031
Genomic classes (GC3 vs GC1/2)	4.14 (1.79-9.54)	0.0009
Multivariable model 3 (n=426, 35 events)		
Stage (T1/CIS vs Ta)	3.39 (1.59-7.24)	0.002
Grade (high vs low)	0.79 (0.35-1.78)	0.57
Genomic classes (GC3 vs GC1/2)	3.44 (1.57-7.51)	0.002
Multivariable model 4 (n=530, 65 events)		
EORTC risk score (>6 vs ≤6)	3.57 (2.06-6.18)	5.9×10^{-6}
Transcriptomic class (2a/2b vs 1/3)	2.55 (1.27-5.10)	0.008
Multivariable model 5 (n=502, 62 events)		
EAU risk score (High vs intermediate/low)	2.38 (1.32-4.31)	0.004
Transcriptomic class (2a/2b vs 1/3)	3.51 (1.65-7.48)	0.001
Multivariable model 6 (n=530, 65 events)		
Stage (T1/CIS vs Ta)	2.88 (1.65-5.02)	0.0002

Grade (high vs low)	1.74 (0.97-3.13)	0.07
Transcriptomic class (2a/2b vs 1/3)	2.32 (1.14-4.71)	0.02
Multivariable model 7 (n=530, 65 events)		
EORTC risk score (>6 vs ≤6)	3.24 (1.82-5.77)	6.6×10^{-5}
T1HG subtype (1/3 vs 2)	2.45 (1.45-4.14)	0.008
Multivariable model 8 (n=502, 62 events)		
EAU risk score (high vs intermediate/low)	2.12 (1.14-3.96)	0.018
T1HG subtype (1/3 vs 2)	3.27 (1.93-5.54)	1.14×10^{-5}
Multivariable model 9 (n=530, 65 events)		
Stage (T1/CIS vs Ta)	2.74 (1.55-4.82)	0.0004
Grade (high vs low)	1.63 (0.89-3.01)	0.11
T1HG subtype (1 vs 2+3)	2.27 (1.32-3.92)	0.003
Multivariable model 10 (n=660, 54 events)		
EORTC risk score (>6 vs ≤6)	3.55 (1.90-6.64)	7.5×10^{-5}
Progression signature, qPCR (high vs low)	2.57 (1.34-4.96)	0.005
Multivariable model 11 (n=619, 51 events)		
EAU risk score (high vs intermediate/low)	2.87 (1.37-6.02)	0.005
Progression signature, qPCR (high vs low)	3.18 (1.60-6.32)	0.001
Multivariable model 12 (n=660, 54 events)		
Stage (T1/CIS vs Ta)	3.27 (1.73-6.17)	0.0003
Grade (high vs low)	1.43 (0.75-2.72)	0.27
Progression signature, qPCR (high vs low)	2.33 (1.19-4.58)	0.014
Recurrence-free survival		

	HR (95% CI)	p-value
Univariate analysis		
Clinical features (n=629, 410 events)		
Age	1.02 (0.98-1.07)	0.35
Gender (male vs female)	0.81 (0.64-1.01)	0.06
Stage (T1/CIS vs Ta)	1.05 (0.84-1.32)	0.64
Grade (high vs low)	1.13 (0.93-1.38)	0.23
Concomitant CIS (yes vs no)	1.74 (1.36-2.23)	0.00001
EORTC risk score (>6 vs ≤6)	1.19 (0.98-1.46)	0.08
EAU risk score (High vs. intermediate/low; n=597, 396 events)	1.19 (0.97-1.45)	0.09
Smoking history (current/former vs never; n=483, 317 events)	1.33 (0.97-1.82)	0.07
RNA-Seq features(n=511, 348 events)		
Transcriptomic class 2a vs 1	1.57 (1.13-2.18)	0.007
Transcriptomic class 2b vs 1	1.48 (1.08-2.02)	0.02
Transcriptomic class 3 vs 1	1.35 (0.95-1.92)	0.1
Progression signature (continuous)	1.69 (1.18-2.43)	0.004
CIS signature (continuous)	0.95 (0.71-1.28)	0.73
T1HG subtype 1 vs 2	1.16 (0.84-1.60)	0.36
T1HG subtype 3 vs 2	0.97 (0.64-1.46)	0.88
Immune score (continuous)	1.00 (0.99-1.01)	0.9
Mutational signatures (n=420, 283 events)		
Signature 1 (above vs below median)	1.18 (0.94-1.49)	0.16
Signature 2 (above vs below median)	0.88 (0.69-1.11)	0.28
Signature 5 (above vs below median)	1.14 (0.90-1.44)	0.28
Signature 13 (above vs below median)	1.03 (0.81-1.30)	0.83
qPCR features (n=537, 339 events)		
Progression signature (high vs low)	1.47 (1.18-1.82)	0.0005

Progression signature (continuous)	1.17 (1.08-1.27)	0.0002
Copy number features (n=399, 239 events)		
Genomic classes (GC3 vs GC1/2)	1.57 (1.21-2.04)	0.0007
Genome altered in Mb (continuous)	1.45 (1.19-1.77)	0.0002
Multivariable model 1 (n=399, 239 events)		
EORTC risk score (>6 vs ≤6)	1.06 (0.79-1.41)	0.69
Genomic classes (GC3 vs GC1/2)	1.53 (1.14-2.05)	0.004
Multivariable model 2 (n=511, 348 events)		
EORTC risk score (>6 vs ≤6)	1.11 (0.88-1.39)	0.35
Transcriptomic class (2a/2b vs 1/3)	1.26 (1.00-1.59)	0.046
Multivariable model 3 (n=511, 348 events)		
EORTC risk score (>6 vs ≤6)	1.09 (0.87-1.38)	0.44
Transcriptomic class 2a vs 1	1.51 (1.07-2.13)	0.02
Transcriptomic class 2b vs 1	1.45 (1.05-1.99)	0.02
Transcriptomic class 3 vs 1	1.34 (0.94-1.91)	0.10
Multivariable model 4 (n=537, 339 events)		
EORTC risk score (>6 vs ≤6)	1.23 (0.98-1.55)	0.07
Progression signature, qPCR (high vs low)	1.37 (1.09-1.71)	0.007

HR = hazard ratio; CI = confidence interval; CIS = carcinoma in situ; EORTC = European Organisation for Research and Treatment of Cancer; EAU = European Association of Urology. Source data are provided as a Supplementary Source Data file.

Supplementary Table 3. Clinical characteristics for patients stratified by transcriptomic class

	Class 1 n (%)	Class 2a n (%)	Class 2b n (%)	Class 3 n (%)	n (%)	*p*-value
Patients	102 (19)	142 (27)	181 (34)	110 (21)	535	
Median age (years)	65	71	70	68		0.002
Gender						0.84
Male	79 (78)	112 (79)	136 (75)	87 (79)	414 (77)	
Female	23 (23)	30 (21)	45 (25)	23 (21)	121 (23)	
Smoking status						0.07
Current	38 (37)	25 (18)	55 (30)	51 (46)	169 (32)	
Former	34 (33)	39 (28)	63 (35)	31 (28)	167 (31)	
Never	16 (16)	16 (11)	18 (10)	12 (11)	62 (12)	
Unknown	14 (14)	62 (44)	45 (25)	16 (15)	137 (26)	
Stage						1.9×10^{-10} [a]
Ta	93 (91)	79 (56)	132 (73)	93 (85)	397 (74)	
T1	9 (9)	61 (43)	48 (27)	17 (16)	135 (25)	
CIS	0	2 (1)	1 (1)	0	3 (1)	
Grade						7.2×10^{-14}
High	25 (25)	92 (65)	75 (41)	23 (21)	215 (40)	
Low	77 (76)	50 (35)	106 (59)	87 (79)	320 (60)	
Concomitant CIS						9.2×10^{-7}
Yes	4 (4)	39 (28)	24 (13)	10 (9)	77 (14)	
No	98 (96)	103 (73)	157 (87)	100 (91)	458 (86)	
Growth pattern						0.003
Papillary	100 (98)	123 (87)	150 (83)	107 (97)	480 (90)	
Mixed	0	5 (4)	5 (3)	0	10 (2)	
Solid	0	6 (4)	7 (4)	0	13 (2)	
Unknown	2 (2)	8 (6)	19 (11)	3 (3)	31 (6)	
Tumor Size						0.047
< 3 cm	73 (72)	97 (68)	109 (60)	67 (61)	346 (65)	
≥ 3 cm	8 (8)	30 (21)	34 (19)	18 (16)	90 (17)	
Unknown	21 (21)	15 (11)	38 (21)	25 (23)	99 (19)	
Incident tumor						0.002
Yes	62 (61)	88 (62)	77 (43)	60 (55)	287 (54)	
No	40 (39)	54 (38)	104 (58)	50 (46)	248 (46)	
BCG treatment after TURB						4.5×10^{-15}
Yes	12 (12)	81 (57)	48 (27)	22 (20)	163 (30)	
No	90 (88)	61 (43)	133 (73)	88 (80)	372 (70)	
BCG/MMC/Chemo ever in disease course						9.4×10^{-9}
Yes	53 (52)	100 (70)	92 (51)	34 (31)	279 (52)	
No	47 (46)	33 (23)	78 (43)	65 (59)	223 (42)	
Unknown	2 (2)	9 (6)	11 (6)	11 (10)	33 (6)	
Progression to MIBC						8.4×10^{-6}
Yes	3 (3)	32 (23)	23 (13)	7 (6)	65 (12)	
No	97 (95)	109 (77)	156 (86)	103 (94)	465 (87)	
Unknown	2 (2)	1 (1)	2 (1)	0	5 (1)	
Mean recurrence rate per year	0.40	1.11	0.67	0.70	0.74	0.0001
EORTC risk score						5.2×10^{-15}
High (>6)	20 (20)	93 (66)	72 (40)	26 (24)	211 (39)	
Low (≤6)	82 (80)	49 (35)	109 (60)	84 (76)	324 (61)	
EAU risk score						6.1×10^{-10}
High	32 (31)	104 (73)	99 (55)	42 (38)	277 (52)	
Intermediate	35 (34)	27 (19)	58 (32)	38 (35)	158 (30)	
Low	24 (24)	9 (3)	15 (8)	24 (22)	72 (13)	
Unknown	11 (11)	2 (1)	9 (5)	6 (5)	28 (5)	

P-values were calculated using a Kruskal-Wallis rank sum test for continuous variables and two-sided Fisher's exact test or chi-square test for categorical variables. CIS = carcinoma *in situ*; BCG = Bacillus Calmette-Guérin; EORTC = European Organisation for Research and Treatment of Cancer; EAU = European Association of Urology. [a]Comparison between Ta and T1+CIS tumors. Source data are provided as a Supplementary Source Data file.

Supplementary Table 4. Clinical characteristics for patients stratified by genomic class

	GC1 n (%)	GC2 n (%)	GC3 n (%)	n (%)	*p*-value
Patients	158 (33)	158 (33)	157 (33)	473	
Median age (years)	67	70	72		2.3×10^{-5}
Gender					0.64
Male	114 (72)	120 (76)	120 (76)	354 (75)	
Female	44 (28)	38 (24)	37 (24)	119 (25)	
Smoking status					0.03
Current	59 (37)	58 (37)	45 (29)	162 (34)	
Former	42 (27)	42 (27)	66 (42)	150 (32)	
Never	27 (17)	25 (16)	18 (12)	70 (15)	
Unknown	30 (19)	33 (21)	28 (18)	91 (19)	
Stage					3.8×10^{-13} [a]
Ta	137 (87)	121 (77)	77 (49)	335 (71)	
T1	18 (11)	36 (23)	77 (49)	131 (28)	
CIS	3 (2)	1 (1)	3 (2)	7 (1)	
Grade					2.5×10^{-21}
High	32 (20)	54 (34)	113 (72)	199 (42)	
Low	126 (80)	104 (66)	44 (28)	274 (58)	
Concomitant CIS					1.3×10^{-9}
Yes	10 (6)	10 (6)	45 (29)	65 (14)	
No	148 (94)	148 (94)	112 (71)	408 (86)	
Growth pattern					0.60
Papillary	150 (95)	140 (89)	132 (84)	422 (89)	
Mixed	1 (1)	2 (1)	4 (3)	7 (1)	
Solid	2 (1)	2 (1)	3 (2)	7 (1)	
Unknown	5 (3)	14 (9)	18 (12)	37 (8)	
Size					0.16
< 3 cm	96 (61)	102 (65)	96 (61)	294 (62)	
≥ 3 cm	15 (9)	23 (15)	29 (19)	67 (14)	
Unknown	47 (30)	33 (21)	32 (20)	112 (24)	
Incident tumor					0.009
Yes	97 (61)	70 (44)	80 (51)	247 (52)	
No	61 (39)	88 (56)	77 (49)	226 (48)	
BCG treatment after TURB					8.6×10^{-6}
Yes	13 (8)	21 (13)	44 (28)	78 (16)	
No	145 (92)	137 (87)	113 (72)	395 (84)	
BCG/MMC/Chemo ever in disease course					0.038
Yes	56 (35)	68 (43)	78 (50)	202 (43)	
No	102 (65)	90 (57)	79 (50)	271 (57)	
Progression to MIBC					0.0005
Yes	6 (4)	6 (4)	23 (15)	35 (7)	
No	132 (84)	136 (86)	123 (78)	391 (83)	
Unknown	20 (13)	16 (10)	11 (7)	47 (10)	
Mean recurrence rate per year	0.41	0.54	0.99	0.65	2.04×10^{-5}
EORTC risk score					1.2×10^{-24}
High (>6)	25 (16)	44 (28)	110 (70)	179 (38)	
Low (≤6)	133 (84)	114 (72)	47 (30)	294 (62)	
EAU risk score					7.7×10^{-17}
High	47 (30)	76 (48)	128 (82)	251 (53)	
Intermediate	55 (35)	53 (34)	18 (12)	126 (27)	
Low	36 (23)	21 (13)	6 (4)	63 (13)	
Unknown	20 (13)	8 (5)	5 (3)	33 (7)	

P-values were calculated using a Kruskal-Wallis rank sum test for continuous variables and two-sided Fisher's exact test or chi-square test for categorical variables. GC = genomic class; CIS = carcinoma in situ; BCG = Bacillus Calmette-Guérin; EORTC = European Organisation for Research and Treatment of Cancer; EAU = European Association of Urology. [a]Comparison between Ta and T1+CIS tumors. Source data are provided as a Supplementary Source Data file.

Supplementary Table 5. Cox regression analysis in independent cohorts

Progression-free survival		
	HR (95% CI)	*p*-value
Univariate analysis		
Clinical features (n=521, 96 events)		
Stage (T1/CIS vs Ta)	2.00 (1.29-3.08)	0.002
UROMOL2021 classes (n=511, 94 events)		
Transcriptomic class 2a vs 1	3.27 (1.91-5.59)	1.6×10^{-5}
Transcriptomic class 2b vs 1	2.39 (1.22-4.07)	0.011
Transcriptomic class 3 vs 1	2.28 (0.99-5.26)	0.052
T1HG subtype (n=512, 95 events)		
T1HG subtype 1 vs 2	1.30 (0.69-2.43)	0.42
T1HG subtype 3 vs 2	2.37 (1.51-3.71)	0.0002
Multivariable model 1 (n=510, 94 events)		
Stage (T1/CIS vs Ta)	1.27 (0.76-2.13)	0.36
Transcriptomic class 2a vs 1	2.84 (1.53-5.27)	0.0009
Transcriptomic class 2b vs 1	2.10 (1.01-4.36)	0.046
Transcriptomic class 3 vs 1	2.23 (0.96-5.14)	0.061
Multivariable model 2 (n=511, 95 events)		
Stage (T1/CIS vs Ta)	1.78 (1.11-2.86)	0.017
T1HG subtype 1 vs 2	0.98 (0.51-1.90)	0.95
T1HG subtype 3 vs 2	1.91 (1.19-3.08)	0.008

HR = hazard ratio; CI = confidence interval; CIS = carcinoma in situ; EORTC = European Organisation for Research and Treatment of Cancer; EAU = European Association of Urology. Source data are provided as a Supplementary Source Data file.

Supplementary Table 6. Gene expression signatures

	Gene list
Early cell cycle	*CCND1, CCND2, CCND3, RBL2, ID1, ID2, ID3, WEE1*
Late cell cycle	*CDK1, CDK4, CDK2, CCNE1, CDC20, CCNB2, CCNB1, CCNA2, BUB1, CDC25A, CCNE2, MYBL2, FOXM1, PLK1*
Uroplakins	*UPK1B, UPK3A, UPK1A, UPK2, UPK3B*
Cancer stem cell markers	*PROM1, ALDH2, ALDH1A1, ALDH1A3, CD47, ALDH1A2, NES, THY1, RPSA, SHH, ITGA6, CD44*
Epithelial-mesenchymal transition	*SOX9, TWIST1, FOXF1, ZEB1, ZEB2, GATA6*
Differentiation	*TMEM163, GATA3, BAMBI, PPARG, GRHL3, SNX31, DHRS2, HPGD, SPINK1, FOXA1, FOXQ1, BHMT, SCNN1G*
FGFR3-coexpressed genes	*C3orf54, CAPNS2, SEMA4B, WNT7B, DUOXA1, C16orf74, ZNF385A, SMAD3, SLC2A9, D4S234E, TP63, CLCA4, IRS1, SYTL1, PLCH2, SSH3, FGFR3, PTPN13, DUOX1, TMPRSS4*
EGFR ligands	*EGFR, AREG, AREGB, EREG, HBEGF, TGFA*
Hypoxia	*CAV1, COL5A1, ITGA5, P4HA2, SLC16A1, TGFBI, DPYSL2, SRPX, TRAM2, SYDE1, LRP1, PDLIM2, SAV1, AHNAK2, CAD, CYP1B1, DAAM1, DSC2, SLC2A3, FUT11, GLG1, GULP1, LDLR, THBS4*
DNA replication	*DNA2, FEN1, LIG1, MCM2, MCM3, MCM4, MCM5, MCM6, MCM7, PCNA, POLA1, POLA2, POLD1, POLD2, POLD3, POLD4, POLE, POLE2, POLE3, POLE4, PRIM1, PRIM2, RFC1, RFC2, RFC3, RFC4, RFC5, RNASEH1, RNASEH2A, RNASEH2B, RNASEH2C, RPA1, RPA2, RPA3, RPA4, SSBP1*
Immune checkpoint	*CD274, CTLA4, HAVCR2, LAG3, PDCD1, PDCD1LG2, TIGIT*
Antigen presenting machinery	*B2M, HLA-A, HLA-B, HLA-C, TAP1, TAP2*
IFN-γ	*TIGIT, CD27, CD8A, PDCD1LG2, LAG3, CD274, CXCR6, CMKLR1, NKG7, CCL5, PSMB10, IDO1, CXCL9, HLA-DQA1, CD276, STAT1, HLA-DRB1, HLA-E*
$CD8^+$ T cells	*CD8A, CXCL10, CXCL9, GZMA, GZMB, IFNG, PRF1, TBX21*
Pan-fibroblast TGF-β	*ACTA2, ACTG2, ADAM12, ADAM19, CNN1, COL4A1, CTGF, CTPS1, FAM101B, FSTL3, HSPB1, IGFBP3, PXDC1, SEMA7A, SH3PXD2A, TAGLN, TGFBI, TNS1, TPM1*
12-gene progression signature	*KPNA2, BIRC5, UBE2C, CDC25B, MSN, COL4A1, COL18A1, COL4A3BP, NEK1, MBNL2, SKAP2, FABP4*
CIS signature	*IL13RA1, FBXL5, ARL5A, CXCR4, F13B, SHOC2, IL6ST, HLA-DQA1, SPOP, EFEMP1, DCN, COL15A1, LYZ, SPARC, IGKC, TCF4, KRAS, SDCBP, COL3A1, FBXW2, PDGFC, SGCE, BIRC2, GAPVD1, FLNA, PPP2R5C, LUM, MBD4, UAP1, TOP2A, RARRES1, CLIC4, KPNA2, FGFR3, LAMB3, ANXA10, CRTAC1, TMPRSS4, CTSE, MST1R, FABP4, CA12, ITGB4, TNNI2, ST3GAL4, PKP1, BCAM, NDUFA4L2, TRIM29, SH3BP1, LTBP3, LYPD3, CDH11, BST2, EEF1A2, CLCA4, BMP7, AKR1B10, KCTD12, KYNU, UPK2, CFD, TMEM45A*

Supplementary Table 7. Gene lists used for the RNA-based estimation of immune cell infiltration

Immune cell population[a]	Gene list
B cells	*BLK, CD19, FCRL2, MS4A1, TNFRSF17, TCL1A, SPIB, PNOC*
Cytotoxic cells	*PRF1, GZMA, GZMB, NKG7, GZMH, KLRK1, KLRB1, KLRD1, CTSW, GNLY*
Dendritic cells	*CCL13, CD209, HSD11B1*
Exhausted T cells	*LAG3, CD244, EOMES, PTGER4*
Macrophages	*CD68, CD84, CD163, MS4A4A*
Mast cells	*TPSB2, TPSAB1, CPA3, MS4A2, HDC*
Neutrophils	*FPR1, SIGLEC5, CSF3R, FCAR, FCGR3B, CEACAM3, S100A12*
NK $CD56^-$ cells	*KIR3DL1, IL21R*
NK cells	*XCL1, XCL2, NCR1*
T cells	*CD68, CD3D, CD3E, SH2D1A, TRAT1, CD3G*
T_H1 cells	*TBX21*
Tregs	*FOXP3*
$CD8^+$ T cells	*CD8A, CD8B*
$CD4^+$ T cells	*IGFBP4, ITM2A, AMIGO2, TRAT1, CD40LG, ICOS*

[a] Gene lists were obtained from [76], except $CD4^+$ T cells which was obtained from [77].

Supplementary Table 8. Gene lists used for regulon analysis

	Gene list
Transcription factors previously associated with bladder cancer (obtained from [33])	*AR, PGR, ESR1, ESR2, PPARG, RARA, RARB, RARG, RXRA, RXRG, ERBB2, ERBB3, FGFR1, FGFR3, EGFR, FOXA1, FOXM1, GATA3, GATA6, HIF1A, KLF4, STAT3, TP63*
Candidate regulators associated with chromatin remodeling in cancer (obtained from [34])	*EGR1, HNF4A, YAP1, FLI1, KDM5B, CREB1, IRF3, DNMT1, DNMT3A, DNMT3B, KAT5, KAT7, KAT6A, KAT6B, KAT2A, KAT2B, EP300, CREBBP, NCOA1, NCOA3, HDAC1, HDAC2, HDAC3, HDAC4, HDAC5, HDAC6, HDAC7, HDAC8, HDAC9, HDAC10, HDAC11, SIRT1, SIRT2, SIRT3, SIRT4, SIRT5, SIRT6, SIRT7, SUV39H1, EHMT2, SETDB2, KMT2A, KMT2B, KMT2C, KMT2D, KMT2E, SETD2, NSD1, SMYD2, SMYD3, NSD3, NSD2, DOT1L, KMT5A, EZH2, PRDM14, CARM1, PRMT5, PRMT6, KDM1A, KDM1B, KDM2A, KDM2B, KDM3A, KDM3B, KDM4A, KDM4B, KDM4C, KDM4D, KDM4E, KDM5A, KDM5C, KDM5D, KDM6A, KDM6B, KDM7A, KDM8, ARID1A*

Supplementary Table 9. Primer sequences for genes in the 12-gene progression signature

Gene	Upstream primer sequence 5′–3′	Downstream primer sequence 5′–3′
BIRC5	5′-CTGAAGTCTGGCGTAAGATGATG-3′	5′-GAAGCTGTAACAATCCACCCTG-3′
CDC25B	5′-GATGGAAGGTTGGATGGATG-3′	5′-ACCTGGTTTGGGTATGCAAG-3′
COL4A1	5′-CTGCCTGGAGGAGTTTAGAAGTG-3′	5′-CTGTAAGCGTTTGCGTAGTAATTG-3′
FABP4	5′-AGAGAAAACGAGAGGATGATAAACTG-3′	5′-CTTATGCTCTCTCATAAACTCTCGTG-3′
KPNA2	5′-GCAGATTTTAAGACACAAAAGGAAG-3′	5′-AAGGTACACAATCTGTTCAACTGTTC-3′
MBNL2	5′-ACTTCATCCAGTGCCCACTTTC-3′	5′-GGGGTTACAGGTGCTAGGTAAGG-3′
MSN	5′-CCTGACCTTGAGGAGTCTTGTG-3′	5′-AATATAGGACATATCACCAAGTGAGC-3′
COL18A1	5′-GGGCTGGTTCTGTAATTGTGTG-3′	5′-AAAAGGTCACTTTTATTTGCCTGTC-3′
COL4A3BP	5′-TTTCTGTGGATCATGACAGTGC-3′	5′-CAAGGTTTGACAAATCATAGCAAC-3′
NEK1	5′-CTAAAAGACCAGCTTCAGGACAAAAC-3′	5′-CTAAAGGTATTCCATATTTAGCGGC-3′
SKAP2	5′-TGGAGATGTATGATATTTGAGAGTCC-3′	5′-CTAAATCCAAAGCATTTGCAGAC-3′
UBE2C	5′-TCTAGGAGAACCCAACATTGATAGTC-3′	5′-TCTTGCAGGTACTTCTTAAAAGCTG-3′
