# Supplementary material for: An integrated multi-omics analysis identifies prognostic molecular subtypes of non-muscle-invasive bladder cancer
Source: Nat Commun. 2021 Apr 16;12:2301. doi: 10.1038/s41467-021-22465-w (PMC8052448; doi:10.1038/s41467-021-22465-w)
Supplement: Supplementary file 3 — Description of Additional Supplementary Files [file 41467_2021_22465_MOESM3_ESM.pdf]

Description of Additional Supplementary Files

File Name: Supplementary Data 1
Description: Antibody panels, dilutions and reagents
File Name: Supplementary Data 2
Description: Normalized gene expression data
File Name: Supplementary Data 3
Description: Processed proteomics data
File Name: Supplementary Data 4
Description: Processed EPIC BeadChip methylation data

File Name: Supplementary Software 1
Description: NMIBC classification
