# Supplementary material for: An integrated multi-omics analysis identifies prognostic molecular subtypes of non-muscle-invasive bladder cancer
Source: Nat Commun. 2021 Apr 16;12:2301. doi: 10.1038/s41467-021-22465-w (PMC8052448; doi:10.1038/s41467-021-22465-w)
Supplement: Supplementary file 8 — Reporting Summary [file 41467_2021_22465_MOESM8_ESM.pdf]

nature research

Corresponding author(s): Prof. Lars Dyrskjøt

Last updated by author(s): Mar 4, 2021

Reporting Summary

Nature Research wishes to improve the reproducibility of the work that we publish. This form provides structure for consistency and transparency in reporting. For further information on Nature Research policies, see our Editorial Policies and the Editorial Policy Checklist.

Statistics

For all statistical analyses, confirm that the following items are present in the figure legend, table legend, main text, or Methods section.

n/a	Confirmed	
☐	☒	The exact sample size (*n*) for each experimental group/condition, given as a discrete number and unit of measurement
☐	☒	A statement on whether measurements were taken from distinct samples or whether the same sample was measured repeatedly
☐	☒	The statistical test(s) used AND whether they are one- or two-sided<br>*Only common tests should be described solely by name; describe more complex techniques in the Methods section.*
☐	☒	A description of all covariates tested
☐	☒	A description of any assumptions or corrections, such as tests of normality and adjustment for multiple comparisons
☐	☒	A full description of the statistical parameters including central tendency (e.g. means) or other basic estimates (e.g. regression coefficient) AND variation (e.g. standard deviation) or associated estimates of uncertainty (e.g. confidence intervals)
☐	☒	For null hypothesis testing, the test statistic (e.g. *F*, *t*, *r*) with confidence intervals, effect sizes, degrees of freedom and *P* value noted<br>*Give P values as exact values whenever suitable.*
☒	☐	For Bayesian analysis, information on the choice of priors and Markov chain Monte Carlo settings
☒	☐	For hierarchical and complex designs, identification of the appropriate level for tests and full reporting of outcomes
☐	☒	Estimates of effect sizes (e.g. Cohen's *d*, Pearson's *r*), indicating how they were calculated

Our web collection on statistics for biologists contains articles on many of the points above.

Software and code

Policy information about availability of computer code

Data collection	No software was used for data collection
Data analysis	SNP data were analyzed using the following software: GenomeStudio 2.0, ASCAT v2.3.<br><br>RNA-Seq data were processed using Salmon v0.10.0 to quantify the amount of each transcript (GRCh38 annotation). Tximport v1.12.3 and edgeR v3.26.8 were used to summmarize the expression at gene-level and normalize the data, respectively. RNA-Seq data were analyzed using the following software/R packages: ConsensusClusterPlus v1.52.0, CancerSubtypes v1.10.0, STAR v2.7, GATK v.4.1.0.0 (PICARD, GATK tools, SplitNCigarReads, BaseRecalibrator, ApplyBQSR, HaplotypeCaller), SnpEff v4.3t, SomaticSignatures v2.20.0, MutationalPatterns v1.10.0, RTN v2.12.1, RTNsurvival v1.12.2, reactome.db v1.68.0, KEGGREST v1.24.1, GSVA v1.32.0, WISP v2.3, survival v3.1.12, survminer v0.4.8, pROC v1.16.2, ggplot2 v3.3.2, reshape2 v1.4.4, tidyverse v1.3.0, ComplexHeatmap v2.4.3.<br>The transcriptomic classifier is available as a web application at http://nmibc-class.dk or as an R package at https://github.com/sialindskrog/classifyNMIBC.<br><br>DNA methylation data were processed using the RnBeads v2.2 R package.<br><br>Quantification of protein markers was carried out using Visiopharm software (Visiopharm A/S, Hørsholm, Denmark).

For manuscripts utilizing custom algorithms or software that are central to the research but not yet described in published literature, software must be made available to editors and reviewers. We strongly encourage code deposition in a community repository (e.g. GitHub). See the Nature Research guidelines for submitting code & software for further information.

Data

Policy information about availability of data

All manuscripts must include a data availability statement. This statement should provide the following information, where applicable:

- Accession codes, unique identifiers, or web links for publicly available datasets
- A list of figures that have associated raw data
- A description of any restrictions on data availability

Raw sequencing and SNP data are deposited and available under controlled access at The European Genome-phenome Archive (EGA), which is hosted by the European Bioinformatics Institute (EBI) and the Centre for Genomic Regulation (CRG). The RNA-Seq data are available under accession code: EGAS00001004693 [https://www.ebi.ac.uk/ega/studies/EGAS00001004693] and the SNP data are available under accession code: EGAS00001004862 [https://www.ebi.ac.uk/ega/studies/EGAS00001004862].

The data are available under controlled access at EGA. Due to privacy laws, data will be available following new approvals by ethical committees and data protection agencies. The data release process can be initiated by contacting the corresponding author (lars@clin.au.dk).
Processed normalized mRNA read counts are available in Supplementary Data 2. Source data are provided with this paper. The remaining data are available within the Article file, Supplementary Information or from the authors upon request.

Transcriptomics data from 11 historical cohorts and 3 unpublished cohorts were used for validation: Kim, microarray, GEO: GSE13507 [https://www.ncbi.nlm.nih.gov/geo/query/acc.cgi?acc=GSE13507]; Lindgren, microarray, GEO: GSE32549 [https://www.ncbi.nlm.nih.gov/geo/query/acc.cgi?acc=GSE32549]; Sjödahl2012, microarray, GEO: GSE32894 [https://www.ncbi.nlm.nih.gov/geo/query/acc.cgi?acc=GSE32894]; CIT, microarray, ArrayExpress: E-MTAB-1803 [https://www.ebi.ac.uk/arrayexpress/experiments/E-MTAB-1803/]; Choi, microarray, GEO: GSE48075 [https://www.ncbi.nlm.nih.gov/geo/query/acc.cgi?acc=GSE48075]; Sjödahl2017, microarray, GEO: GSE83586 [https://www.ncbi.nlm.nih.gov/geo/query/acc.cgi?acc=GSE83586]; Song, microarray, GEO: GSE120736 [https://www.ncbi.nlm.nih.gov/geo/query/acc.cgi?acc=GSE120736]; Sjödahl2019, microarray, GEO: GSE128959 [https://www.ncbi.nlm.nih.gov/geo/query/acc.cgi?acc=GSE128959]; Aarhus microarrays, GEO: GSE3167 [https://www.ncbi.nlm.nih.gov/geo/query/acc.cgi?acc=GSE3167] and GSE5479 [https://www.ncbi.nlm.nih.gov/geo/query/acc.cgi?acc=GSE5479]; Meeks 35, RNA-Seq, GEO: GSE154261 [https://www.ncbi.nlm.nih.gov/geo/query/acc.cgi?acc=GSE154261]; unpublished cohort 1 provided by Margaret Knowles, microarray, GEO: GSE163209 [https://www.ncbi.nlm.nih.gov/geo/query/acc.cgi?acc=GSE163209]; unpublished cohort 2 provided by Richard Bryan, RNA-Seq, EGA: EGAS00001004358 [https://ega-archive.org/studies/EGAS00001004358]; unpublished cohort 3 provided by Trine Strandgaard, RNA-Seq, EGA: EGAS00001005050 [https://ega-archive.org/studies/EGAS00001005050].

Field-specific reporting

Please select the one below that is the best fit for your research. If you are not sure, read the appropriate sections before making your selection.

☒ Life sciences ☐ Behavioural & social sciences ☐ Ecological, evolutionary & environmental sciences

For a reference copy of the document with all sections, see nature.com/documents/nr-reporting-summary-flat.pdf

Life sciences study design

All studies must disclose on these points even when the disclosure is negative.

Sample size	Sample size is not based on power calculations but on sample availability
Data exclusions	Patients were excluded from an analysis (RNA-seq, SNP array or multiplex IHC) if a tumor sample were not suitable for the specific platform (low carcinoma cell fraction, low DNA or RNA concentration, fresh frozen material not available, no clinical information). This resulted in the partially overlapping multi-omics analyses.
Replication	Transcriptomic classes were validated in >1200 independent samples. No replication of other experiments was performed.
Randomization	Randomization was not performed because of the study design.
Blinding	Blinding to outcome was not possible because of the study design, and the fact that this study builds on earlier results.

Reporting for specific materials, systems and methods

We require information from authors about some types of materials, experimental systems and methods used in many studies. Here, indicate whether each material, system or method listed is relevant to your study. If you are not sure if a list item applies to your research, read the appropriate section before selecting a response.

Materials & experimental systems

n/a	Involved in the study	
	x	Antibodies
x		Eukaryotic cell lines
x		Palaeontology and archaeology
x		Animals and other organisms
	x	Human research participants
x		Clinical data
x		Dual use research of concern

Methods

n/a	Involved in the study	
x		ChIP-seq
x		Flow cytometry
x		MRI-based neuroimaging

Antibodies

Antibodies used

Anti-CD8; clone:C8/144B; dilution: 1:150; incubation: 32 min; Dako, Agilent; cat#M710301-2; RRID:AB_2075537
Anti-CD3; clone:2GV6; dilution:Ready to use; incubation:24 min; Ventana Medical Systems, Inc.;cat#790-4341; RRID:NA
Anti-FOXP3; clone:SP97; dilution: 1:10; incubation:32 min;Thermo Fischer; cat#MA5-16365; RRID:AB_2537884
Pan Cytokeratin; clone:AE1/3; dilution: 1:100; incubation:16 min; Dako,Agilent; cat#GA005361-2; RRID:NA
Anti-CD163; clone:MRQ-26; dilution:Ready to use ; incubation:32 min; Ventana Medical Systems, Inc.; cat#;790-4341; RRID:NA
Anti-CD68 PG-M1; clone:PG-M1; dilution:1:100; incubation:32 min; Dako, Agilent; cat#GA61361-2; RRID:AB_2074844
Anti-CD20; clone:L26; dilution:Ready to use; incubation:32 min; Ventana Medical Systems, Inc.; cat#760-2531; RRID:NA
HLA-A,B,C; clone:EMRB-5; dilution:1:100; incubation:32 min; Abcam; cat#ab70328; RRID:AB_1269092
anti-mouse-HRP; Kit:OmniMap anti-Ms HRP (RUO), DISCOVERY; dilution: Ready to use; incubation:12 min; Ventana Medical Systems, Inc.; cat#760-4310
anti-rabit-HRP; Kit:OmniMap anti-Rb HRP (RUO), DISCOVERY; dilution: Ready to use; incubation:16 min; Ventana Medical Systems, Inc.;cat#760-4311
PD-L1;Clone:Sp263; dilution:Ready to use; incubation:60 min; Ventana Medical Systems, Inc.; Cat#790-4905; RRID:AB_2819099
PD-1;Clone:NAT105; dilution:Ready to use; incubation:32 min; Ventana Medical Systems, Inc.; Cat#760-4895; RRID:NA
Pan Cytokeratin; Clone:AE1/3; dilution:1:100; incubation:16 min; Dako, Agilent; Cat#GA005361-2; RRID:NA
GATA3; Clone:L50-823; dilution:Ready to use; incubation:24 min; Ventana Medical Systems, Inc.; Cat#7107749001; RRID:NA
CK5/6; Clone:D5/16 B4; dilution:1:100; incubation:24 min; Dako, Agilent; Cat#M7237; RRID:AB_2281083

Validation

Staining was performed at the Department of Pathology, Aarhus University Hospital on the Discovery ULTRA Staining instrument by a trained technician. Prior to multiplex staining each antibody was stained individually with a chromogenic detection method (DAB) to test specificity according to manufactures guidelines. Tonsil, spleen or lymph node tissue was used as a control tissue.

We have provided a link for the relevant data sheet for each antibody. The data sheet includes the manufacturer's validations statements, quality control procedures and relevant citations:
Anti-CD8; https://www.agilent.com/cs/library/packageinsert/public/108007002.PDF
Anti-CD3; https://pim-eservices.roche.com/eLD/api/downloads/49729da6-7333-ea11-fa90-005056a772fd?countryIsoCode=dk
Anti-FOXP3; https://www.thermofisher.com/order/genome-database/dataSheetPdf?producttype=antibody&productsubtype=antibody_primary&productId=MA5-16365&version=121
Pan Cytokeratin; https://www.agilent.com/cs/library/packageinsert/public/P02066DK_03.pdf or https://www.agilent.com/en/product/immunohistochemistry/antibodies-controls/primary-antibodies/cytokeratin-(dako-omnis)-76170#productdetails
Anti-CD163; https://www.e-labeling.eu/CMC44370030/61057/EN#remarkPopup
Anti-CD68 PG-M1; https://www.agilent.com/en/product/immunohistochemistry/antibodies-controls/primary-antibodies/cd68-(dako-omnis)-76227
Anti-CD20; https://pim-eservices.roche.com/eLD/api/downloads/50d4b69b-6833-ea11-fa90-005056a772fd?countryIsoCode=dk
HLA-A,B,C; https://www.abcam.com/hla-class-1-abc-antibody-emr8-5-ab70328.html
anti-mouse-HRP; https://pim-eservices.roche.com/eLD/api/downloads/2054601c-1513-ea11-fa90-005056a772fd?countryIsoCode=dk
anti-rabit-HRP; https://pim-eservices.roche.com/eLD/api/downloads/2054601c-1513-ea11-fa90-005056a772fd?countryIsoCode=dk
PD-L1;Clone: https://diagnostics.roche.com/global/en/products/tests/ventana-pd-l1-_sp263-assay1.html#productInfo
PD-1;Clone: https://pim-eservices.roche.com/eLD/web/dk/da/products/RTD001087?searchTerm=760-4895&catalog=HealthcareProfessional&orderBy=Relevance
Pan Cytokeratin; https://www.agilent.com/cs/library/packageinsert/public/107609005.PDF
GATA3; https://www.e-labeling.eu/CMC48970010/61172/EN#remarkPopup
CK5/6; https://www.agilent.com/en/product/immunohistochemistry/antibodies-controls/primary-antibodies/cytokeratin-5-6-(concentrate)-76644#literature

Human research participants

Policy information about studies involving human research participants

Population characteristics	UROMOL is a European multicenter study of NMIBC and includes both incident and prevalent early-stage bladder tumors. Covariate-relevant population characteristics includes: age, sex, disease stage, outcome, treatment.
Recruitment	Patient recruitment was carried out in each involved clinical center following national ethics guidelines (see below). As frozen materials were needed for analysis, a bias towards patients with larger tumors where adequate materials for both research and pathological evaluation is expected.
Ethics oversight	Patients in the discovery cohort were included in the UROMOL project and followed according to national guidelines. Further details regarding samples, procedures and clinical follow-up are listed in reference 8. Informed written consent to take part in research projects was obtained from all patients, and all ethical regulations for work with human participants were followed. The study was approved by the Central Denmark Region Committees on Biomedical Research Ethics (#1994/2920; Skejby, Aalborg, Frederiksberg); the Danish National Committee on Health Research Ethics (#1906019), the ethics committee of the University Hospital Erlangen (#3755); the ethics committee of the Technical University of Munich (#2792/10); Medical Ethics Committee of Erasmus MC (MEC#168.922/1998/55; Rotterdam); the Uppsala Region Committee on Biomedical Research Ethics (#2008/252); the Ethical Committee of Faculty of Medicine, University of Belgrade (#440/VI-7); the Ethics Committee (CEIC) of Institut Municipal d'Assistència Sanitària/Hospital del Mar (2008/3296/I); the ethics committee of the University Hospital Jena (#4774-4/16).

Note that full information on the approval of the study protocol must also be provided in the manuscript.
